# Involvement of a small GTP binding protein in HIV-1 release

**DOI:** 10.1186/1742-4690-2-48

**Published:** 2005-08-04

**Authors:** Gilles Audoly, Michel R Popoff, Pablo Gluschankof

**Affiliations:** 1 Unité des Rickettsies, CNRS UMR6020, Faculté de Médecine, 27 bd Jean Moulin, 13385 Marseille cedex 05, IFR48, France; 2 Unité des Bactéries Anaérobies et Toxines, Institut Pasteur, 28 rue du Dr. Roux, 75724 Paris Cedex 15, France

## Abstract

**Background:**

There is evidence suggesting that actin binding to HIV-1 encoded proteins, or even actin dynamics themselves, might play a key role in virus budding and/or release from the infected cell. A crucial step in the reorganisation of the actin cytoskeleton is the engagement of various different GTP binding proteins. We have thus studied the involvement of GTP-binding proteins in the final steps of the HIV-1 viral replication cycle.

**Results:**

Our results demonstrate that virus production is abolished when cellular GTP binding proteins involved in actin polymerisation are inhibited with specific toxins.

**Conclusion:**

We propose a new HIV budding working model whereby Gag interactions with pre-existing endosomal cellular tracks as well as with a yet non identified element of the actin polymerisation pathway are required in order to allow HIV-1 to be released from the infected cell.

## Background

The final step in HIV-1 replication cycle is the release of nascent viral particles from the infected cell. In this way, HIV-1 acquires its lipid bilayer envelope by budding through the plasma membrane of infected T CD4^+ ^cells. The only necessary and sufficient viral element for this event to take place is the expression product of the *gag *gene; i.e. the Pr55gag precursor [1]. Cells only expressing Pr55gag are able to produce and release vesicles, called viral-like particles (VLP), of size and morphology resembling those of immature viral particles [2,3]. A discrete functional sequence, referred to as the L domain encoded by a PTAP motif in the C-terminal, p6 portion of the Gag precursor, catalyses the pinching off of virus particles from the plasma membrane. Indeed, as demonstrated by EM, virus harbouring a modified L domain have been observed to remain attached to the cell via a thin tether [4]. Further work has shown that the interaction between this viral domain and the cellular cytosolic Tsg101 (the tumor susceptibility gene) molecule, that functions in the biogenesis of the multivesicular body (MVB) endosomal compartment [5], is critical for nascent virus detachment from the plasma membrane of the infected T cell [reviewed in 6].

The biological mechanism involved in the production of either a vesicle or an enclosed membrane surrounded virion through membrane budding, implies plasma membrane curvation prior to phospholipid bilayer fusion. Plasma membrane dynamics are partially governed by actin nucleation, a phenomenon in which several cytosolic molecules, such as small GTP binding proteins among others, are involved [7]. Interestingly, GTP binding protein-dependent actin nucleation, is also a key molecular mechanism in endosomal related vesicular transport [reviewed in 8].

Previous studies reported that HIV-1 release from infected cells could be blocked by disturbing the actin network with specific toxins as Cytochalasin D (Cyto D) or Mycalolyde B [9,10]. The published data shows that, although structural viral proteins are transported and localized to the inner face of the plasma membrane in Cyto D treated cells, HIV-1 virions remain attached to the cell, presenting the same phenotype as observed for L-domain mutated viruses [9].

Since actin dynamics are involved in intracellular vesicular transport, and multiple actin nucleation events at the cell cortex lead to the formation of a dense branched filament network that pushes the membrane forward [11], we postulated that the actin polymerisation pathway itself may play a crucial role in efficient HIV-1 release.

## Results

### Inhibition of small GTP-binding proteins abolishes HIV budding

**Figure 1:**
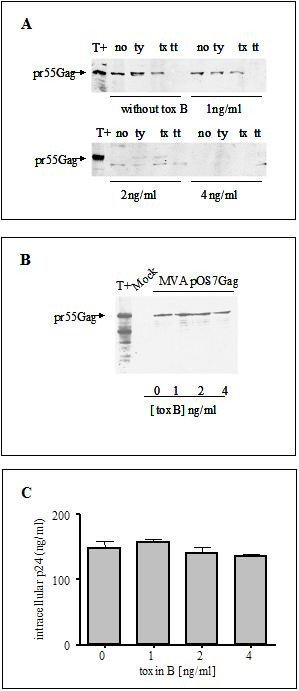
Toxin B inhibits VLP release. A. Toxin B dose dependent inhibition of VLP production. Supernatants of MVA infected/HIV-1 Gag transfected HeLa-CD4 cells, treated or not with various concentrations of Toxin B for 16 h, were clarified by low speed centrifugation and treated or not (no) with Trypsin (ty), Triton X-100 (tx) or with Triton X-100 and Trypsin (tt). VLPs were recovered by centrifugation and subjected to Western Blot analysis. B. Lysates of cells from panel A were subjected to Western Blot analysis. (mock: cells transfected with the vector without any insert, T+: is a cellular extract of HIV-1_NDK _infected H9 cells). C. p24 antigen was quantified in lysates from panel A by ELISA.

We have tested the involvement of plasma membrane related small GTP binding proteins in virus release, using specific bacterial toxins. Toxin B from *Clostridium difficile *inhibits Cdc42, Rho and Rac molecules by modifying the protein structure through threonine glucosylation [12]. This modification blocks their ability to bind downstream effectors, resulting in actin network disruption. We first asked whether or not Toxin B treatment would interfere with Gag budding and release in a system, where high levels of HIV-1 Pr55gag, as the only viral protein, would be produced. Expression of the HIV-1 Gag precursor, by HeLa-CD4 cells, resulted in VLPs released to the media (Fig. 1A and Material and Methods). The fact that VLP related Pr55gag was neither degraded by Trypsin treatment nor disassembled by Triton X-100 detergent addition, strongly suggested that the viral protein might be surrounded by a lipid bilayer (Fig. 1A). Total degradation of Pr55gag was only obtained after Trypsin treatment of detergent solubilized material (Fig. 1A). Incubation of HIV-1 Gag expressing HeLa-CD4 cells with increasing amounts of Toxin B did not induce cell death, since more than 95% of treated cells excluded the Trypan blue dye. Interestingly, VLP release was inhibited in a dose dependent manner with a maximum effect at a Toxin B concentration of 4 ng/ml (Fig. 1A). Conversely, the overall intracellular Gag production was not significantly modified in these experimental conditions, as shown by p24 quantification and western blot analysis of the soluble fraction of detergent lysed treated cells (Fig. 1B, C). These results show that Pr55gag release was abolished when small GTP binding proteins such as Cdc42, Rac, and/or Rho were inhibited.

**Figure 2:**
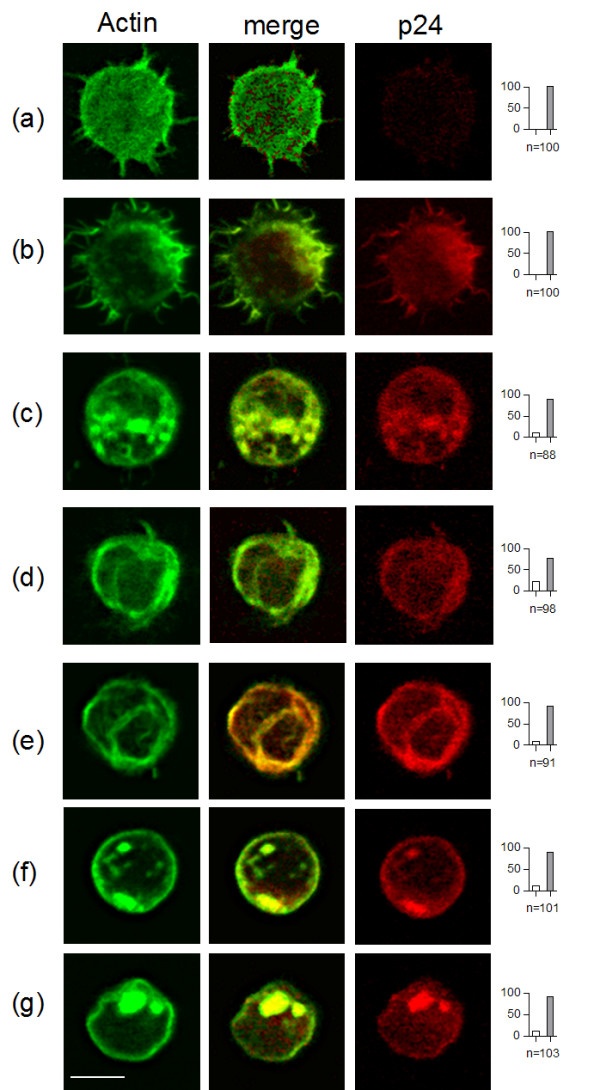
Actin polymerisation and intracellular Gag distribution under toxin treatments. HIV-1 or mock infected Jurkat cells treated or not with 0.5 μg/ml toxins for 16 hours, were stained with Phalloidin-FITC (green) and p24 (red), in order to visualize actin organization and Gag distribution, respectively. A field of about 100 cells was studied for each condition, and the percentage of cells presenting disrupted (grey bar) or not (white bar) cortical actin pattern is represented as an histogram. "n": number of counted cells in the field. a) mock infected cells, b-g) HIV-1_NDK _infected cells. Untreated cells (a-b), cells treated with toxin B (c), LT (d), C3 (e), Cyto D (f), and Iota (g). Bar scale = 10 μm.

In order to define if this is also the case in HIV-1 infected cells, we tested the inhibition of virus production from HIV-1_NDK _infected Jurkat cells in the presence of Toxin B, exoenzyme C3 from *Clostridium botulinum*, and Lethal Toxin 82 (LT) from *Clostridium sordellii*. Exoenzyme C3 ADP-ribosylates specifically Rho proteins, whereas LT glucosylates specific Thr residues from Ras, Rap, Rac and Ral proteins [12,13]. The human Jurkat T cell line was infected with HIV-1_NDK _and maintained 4 days in culture. After washing 3 times with PBS to ensure elimination of previously produced viral particles, cells were grown for another 20 h in complete medium (RPMI) in the presence or absence of increasing amounts of toxins. The highest toxin concentration used corresponds to the maximal sub-lethal toxin concentration, defined as the maximal toxin amount that did not kill the cell (observed by Trypan blue exclusion) in our experimental system. Under these conditions, toxin activity was confirmed by loss of diffused cortical actin as well as actin aggregate formation, monitored by immunofluorescence microscopy on Phalloidin-FITC treated cells. Cellular morphological changes characterized by cell rounding and loss of numerous filopodial projections was also observed (Fig. 2). Gag and actin co-localized both in treated and untreated cells (Fig. 2b–g). Whereas both proteins were exclusively seen in membrane protrusions in infected untreated cells (Fig 2b), cortical actin disorganisation induced changes in HIV-1 Gag distribution in toxin treated cells (Fig. 2c–g).

**Figure 3:**
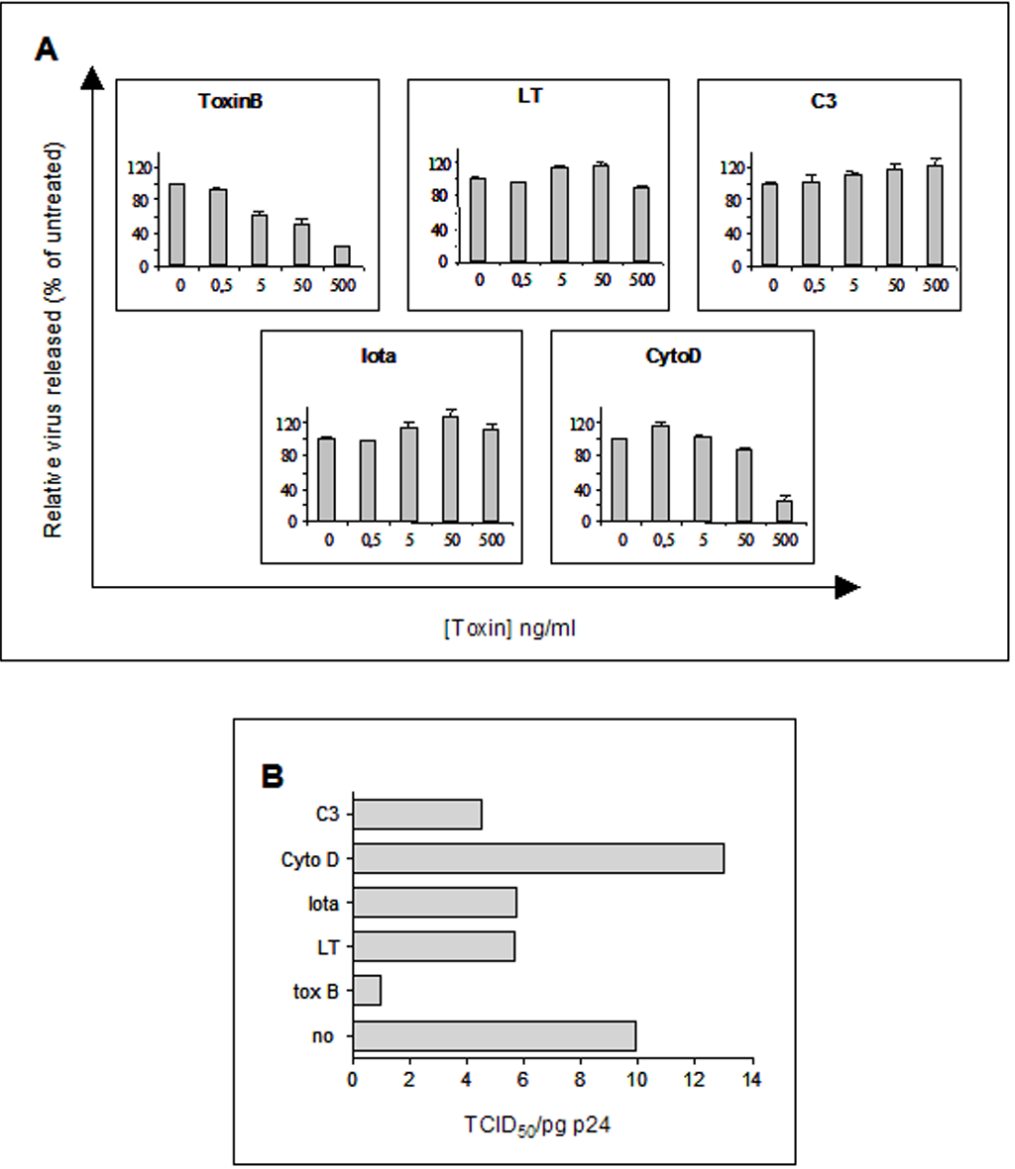
Engagement of small GTP binding proteins in HIV-1 release. Jurkat HIV-1 infected cells were incubated for 20 h with various concentrations of bacterial toxins and Cyto D. A) Clarified supernatants of the culture medium were harvested for p24 quantification by ELISA. Vertical axis indicates the relative HIV-1 production expressed as a percentage of the p24 antigen in the absence of toxin treatment. B) Titres of infectious virus (TCID_50_) released/pg of p24 from the highest toxin concentration dose from infected cells shown in panel A. Data presented corresponds to one out of three independent experiments. Each experiment was performed in triplicate.

We further analysed the viral production capacity of HIV infected T cells treated with the bacterial toxins. Cell culture supernatants of toxin treated or untreated cells were harvested, and intracellular as well as extra cellular p24 antigen was quantified. The intracellular amount of p24 antigen was found to be identical for all cells; i.e. 27.1+/- 1.9 ng p24/10^5 ^cells. The release of p24 was unaffected by C3 and LT but was drastically inhibited by Toxin B (Fig. 3A). These data strongly suggest that indeed active small GTP binding proteins are necessary for HIV-1 to be released from the infected target cell.

The increased amount of Toxin B required to inhibit VLP formation in HeLa cells compared to that required to abolish virus release in Jurkat cells (Fig. 1A and 3A) is due to the susceptibility of each cell line to the action of the toxin.

Unexpectedly, when infected Jurkat cells were incubated in the presence of two different actin disrupting agents, Cyto D or Iota Toxin, only Cyto D inhibited HIV production (Fig. 3A), as already reported [9], whereas Iota toxin did not. (fig 3A). Overnight incubation of HIV-1 infected Jurkat cells with various concentrations of these toxins did not induce cell death (as defined by Trypan blue exclusion) and resulted in toxin-dependent actin depolymerisation, as observed by immunofluorescence microscopy on Phalloidin-FITC treated cells (Fig. 2f, g). Since Cyto D reacts with elongating membrane interacting actin [14], whereas Iota sequesters soluble actin monomers [12], our result suggests that active nucleation at the plasma membrane may be necessary for HIV production.

### Inhibition of small GTP-binding proteins reduces infectivity of HIV-1 particles

We further investigated whether toxin treatments of HIV-1 producing cells had any effect on the infectivity of the *de novo *synthesized virions. Infectivity released into the culture media at the highest toxin concentration used in the experiment represented in figure 3A, was quantified by measuring the TCID_50_/p24 value of supernatants, as described elsewhere [15] (Fig. 3B). Whereas Toxin B lowered the TCID_50_/p24 value of supernatants with a Toxin treated/Toxin untreated TCID_50_/p24 ratio of about 0.1, Cyto D only affected virus infectivity by a factor of 1.3 (Fig. 3B). This suggests that the infectivity of the small amount of released virus from Cyto D treated cells remained almost unchanged. Unexpectedly although C3, Iota and LT did not alter p24 release from infected cells (Fig. 3A), they reduced by about two-fold (Toxin treated/Toxin untreated TCID_50_/p24 ratio ranging from 0.40 to 0.55) the infectivity of cell-free virus (Fig. 3B). This suggests that the status of the actin network in virus-producing cells is relevant for the quality of the virus released into the medium.

**Figure 4:**
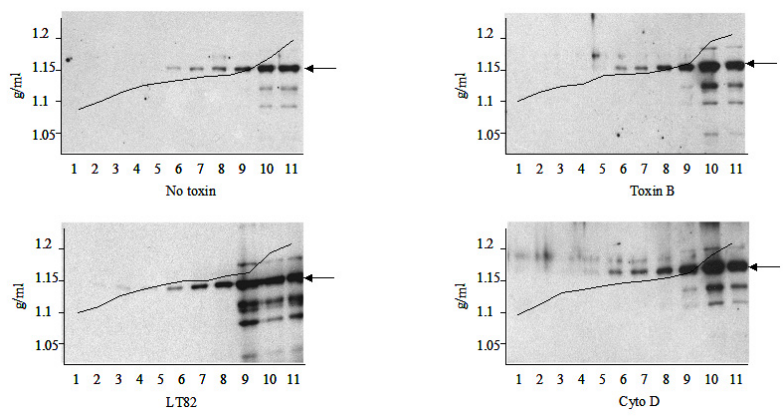
Toxin treatment does not affect intracellular HIV-1 Gag assembly. Non denaturing cytoplasmic lysates of HIV-1_NDK _Jurkat infected cells treated or not with 0.5 μg/ml of Toxin B, LT, or Cyto D for 16 h, were centrifuged through a discontinuous sucrose gradient. Eleven fractions were collected from top to bottom, concentrated by high speed centrifugation, and analysed by Western Blot. Vertical axis shows the sucrose density fractionation in g/ml. Arrows indicate Pr55gag migration.

It is well documented that Gag assembly in the cytoplasm of infected T cells is required as a key step prior to virus budding [16]. Thus, the inhibition of virus production by the action of toxins (Fig 3) could occur at the Gag assembly level rather than at the level of an interaction between plasma membrane actin polymerisation and the viral protein. In order to rule out this possibility, we studied the assembly status of soluble cytoplasmic Pr55gag in toxin treated cells by sucrose gradient analysis as already reported [17]. HIV-1 infected Jurkat cells treated or not with toxins were lysed in non denaturing conditions and the resulting soluble fraction was loaded on a discontinuous sucrose gradient (see Material and Methods section). In all cases, Pr55gag was recovered in fractions 9–11, at a relative density of about 1.15–1.20 g/ml (Fig. 4), corresponding to assembled non-enveloped Gag structures [18]. Thus, the observed toxin dependent inhibition of virus production was indeed at the level of virus release, and not a result of a modification of intracellular events leading to Pr55gag assembling.

## Discussion

In infected and transfected cells the HIV Gag precursor is known to be targeted to the inner face of the plasma membrane and to co-localise with actin. In our study we have shown in Jurkat T-cells that this co-localisation takes place in membrane protrusions (Fig. 2b), as previously shown for SupT1 HIV-1 infected cells [19]. Interestingly, incubation of HIV infected Jurkat cells with the toxins that induced cortical actin disorganisation, produced changes in HIV-1 Gag distribution (Fig. 2c–g). This result reinforces the previously reported physical interaction occurring between the Gag precursor and actin [20-23], and argues, as in Sasaki et al. [10], for a potential role for actin dynamics in Pr55gag intracellular localisation.

We have found that disturbing cortical actin dynamics inhibited virus production [Fig. 3A]. This was observed either by modifying the polymerising actin itself, by Cyto D action, or by inhibiting one key GTP binding protein involved in a molecular pathway that leads to actin nucleation, by Toxin B action. Some GTP binding proteins have been shown to govern actin dynamics as well as intracellular vesicular trafficking [8,24]. Since the viral Gag precursor does not travel through the secretory pathway [1] it is reasonable to hypothesize that HIV virus budding and actin polymerisation through activation of a GTP binding protein may be linked. What is thus the molecular mechanism that can explain this observation? We found that Toxin B abolished HIV-1 production whereas C3 and LT did not. Knowing the spectrum of the toxins targets [12,13], it can be inferred that Cdc42 might be a putative cellular partner to virus release. Cdc42 has been found to be specifically down-regulated in cells latently infected with HIV, suggesting an important role for active Cdc42 in virus infection [25]. It can thus be argued that active Cdc42 may induce an actin polymerisation pathway and allow virus budding and release. Analysis of virus production from HIV infected cells harbouring inactive forms of the Cdc42 molecule should help to ultimately define its involvement in this event.

Our study concluded that C3, Iota and LT reduced infectivity of virus produced. However these toxins did not alter total virus production (Figure 3A and 3B). This suggests that the capacity of the budding viral particle to infect a new target cell is modified through disruption of the actin web of the infected cell. How can actin be then correlated to infection in this particular case? The most possible explanation is based on the budding event itself. HIV selectively incorporates cellular membrane proteins, that have been suggested to be involved in virus infectivity [26], while budding from lipid raft domains at the plasma membrane of the infected cell [27] where the Gag precursor is mainly localised [28]. Since disruption of actin filaments modifies the protein content of lipid rafts [29], the action of the studied toxins on the infected cell might modify the cellular protein content of the lipid raft. HIV may then bud as a virus lacking a cellular component, or harbouring an inhibitory cellular molecule.

Virus entry, by a membrane fusion mechanism, requires actin nucleation [30] through activation of Rac-1 but not Cdc42 or Rho proteins [31]. According to our results actin network remodelling would be a key process for HIV replication, since it will play a crucial role in both early (entry) and late (budding) infectious events, by involvement of different sets of cellular GTP binding proteins.

## Conclusion

We have shown that inhibiting small GTP binding proteins involved in cortical actin dynamics disrupts virus release. This is not the simple consequence of actin network disorganisation since the action of LT, C3 and Iota did not affect virus production. Our results suggest that the actin polymerisation process, potentially via Cdc42 is involved in the final step of the HIV replication cycle.

Analysis of recently published results shows that the implication of intracellular protein transport pathways to late endosomal compartments (i.e. the multivesicular bodies compartment) acts as pre-existing cellular "tracks" for the viral Gag protein-induced budding [32-34]. The data presented here argues for a more complex working model whereby in addition to using an intracellular "track", HIV requires the specific exploitation of actin dynamics in order to be released from the infected cell. Further experimental studies should be done to define the actin activation pathway used by Gag and the chronology of the molecular events involved.

## Materials and methods

### Cell culture and transfection

C8166 and Jurkat cells were grown in RPMI 1640 medium, and HeLa-CD4 cells in MEM medium. Both media were completed with 10% FCS, 2 mM glutamine and 100 U/ml of penicillin-streptomycin.

Cells were infected with the Ankara strain/T7 RNA polymerase (MVA) [35,36] at 1 pfu/cell, 30 min before being transfected by fugene-6 (Roche, Basel, Switzerland) with pos7 vector [36] or recombinant pos7-HIV-1Gag [37].

### VLP analysis

Supernatants of cells were harvested and clarified by low speed centrifugation 24 h after transfection, and released VLP were concentrated by centrifugation at 100,000 × g at 4°C through a 20% sucrose cushion. The resulted pellet was resuspended in TNE and treated or not with 5 μg/ml trypsine and /or 1% Triton X-100. Treated or mock-treated VLPs were resolved on SDS 10% polyacrylamide gel and transferred onto nitrocellulose membrane. Immunoblotting was carried out with human polyclonal IgG purified from HIV-1 positive individuals (HIVIg), followed by peroxydase-conjugated anti-human antibodies incubation. HIV related proteins were detected using the ECL kit (Amersham Biosciences, Upsala, Sweden).

### Cell lysis and density gradient

Cytoplasmic lysates of 5*10^5 ^cells were fractionated according to Gorvel et al. [38] with some modifications. Briefly, cells were washed in PBS and resuspended in 0.5 ml of cold homogenisation buffer (HB) (250 mM sucrose, 3 mM imidazole, 0.1% gelatin) completed with the protease inhibitors cocktail (from Roche). Cell lyses was obtained through 2 cycles of freezing and thawing. The lysates were then clarified by centrifugation and the resultant post nuclear supernatants (PNS), were diluted to 1 ml to obtain a final concentration of 32 % sucrose. A discontinuous sucrose gradient was set up, from bottom to top, as follows: 0.3 ml 62% sucrose, 0.3 ml 45% sucrose, 0.3 ml 35 % sucrose, 1 ml of diluted PNS, 0.6 ml 30% sucrose, 0.6 ml 25% sucrose, 0.6 ml 20 % sucrose, and centrifuged for 1 hr at 100,000 × g. Twelve fractions were collected from top to bottom. An aliquot of each fraction was used to determine the density by measuring the refraction index with a refractometer. Each fraction was diluted 1:3 in TNE buffer (10 mM Tris-HCl buffer pH 7, 0.1 M NaCl, 1 mM EDTA) and the assembled Gag protein was recovered as a pellet, after concentration by high speed centrifugation at 70,000 × g for 30 min. The pellets were resuspended in Laemmli loading buffer, and submitted to SDS 10% PAGE prior to Western Blot analysis.

### Toxins

All toxins used in this study, but Cyto D, were purified as in [39-41]. Cyto D was purchased from Sigma (France).

### Cell infection

Jurkat cells were infected with HIV-1_NDK _at an MOI of 0.5 and maintained 4 days in culture at 5 × 10^5 ^cell/ml. After 3 washes in PBS the cells were grown for another 20 h in complete medium containing serially diluted bacterial toxins. Quantification of viral production by HIV-1 p24 ELISA (Organon Teknika, Boxtel, NL) was done on supernatants, previously clarified by centrifugation at 1500 × g for 5 min. TCID_50 _was determined on C8166 T-lymphocytes as previously described [20].

### Immunofluorescence studies

Cells were incubated on polylysine-covered slides at room temperature for 15 min and immediately fixed in phosphate-buffered saline (PBS) (pH 7.4) containing 3.7% para-formaldehyde and 0.025% glutaraldehyde for 10 min. Fixed cells were treated 10 min in 0.1 M glycine before being permeabilized in PBS containing 0.1% Saponine for 10 min. After two washes in PBS, cells were incubated with 1% bovine serum albumin in PBS (pH 7.4) for 20–30 min. Immunofluorescence staining was performed with phalloidin-FITC (Sigma Aldrich, France) and monoclonal anti p24 (Dako, France) followed by TRITC-labeled anti-mouse antibody (Jakson). The specimens were analysed on a fluorescence microscope. Separate images were taken in the corresponding channels, and merge images were composed. Image acquisition and data processing for all the samples were performed under the same conditions.

## List of abbreviations

VLP : viral-like particles, tsg101 : the tumor susceptibility gene, MVB : the multivesicular body endosomal compartment, Cyto D : Cytochalasin D, LT : Lethal Toxin 82.

## Competing interests

The author(s) declare that they have no competing interests.

## Authors' contributions

GA performed the experiments. PG and GA participated in the experimental design, data interpretation and writing of the manuscript. MP was involved in the interpretation of toxin based experiments
